# Application of unmanned aerial vehicles in modern battlefields and their impact on post‐traumatic stress disorder in military personnel: A review

**DOI:** 10.1002/gps3.70050

**Published:** 2026-08-27

**Authors:** Yu Xue, Yufang Sun, Yongxian Zhang, Yongliang Zhang, Chenglin Sang, Dawei Zhang

**Affiliations:** ^1^ Department of Medical Service The 960th Hospital of the PLA Joint Logistics Support Force Jinan Shandong China; ^2^ Department of Endocrinology The 960th Hospital of the PLA Joint Logistics Support Force Jinan Shandong China; ^3^ Department of Orthopedics The 960th Hospital of the PLA Joint Logistics Support Force Jinan Shandong China; ^4^ The Hospital of Unit 94543 Chinese People's Liberation Army Jining Shandong China; ^5^ Department of Burn and Plastic The 960th Hospital of the PLA Joint Logistics Support Force Jinan Shandong China

**Keywords:** battlefield applications, military mental health, moral injury, neurobiological mechanisms, post‐traumatic stress disorder, unmanned aerial vehicles

## Abstract

The widespread adoption of unmanned aerial vehicles (UAVs) in military operations has profoundly transformed modern warfare, introducing new combat paradigms that significantly affect the psychological well‐being of service members. This review systematically examines the primary applications of UAVs on the battlefield and explores the complex relationship between UAV warfare and post‐traumatic stress disorder (PTSD) among military personnel. It analyses the unique psychological stressors associated with UAV operations, such as the moral dilemmas of remote killing, the persistent tension from continuous surveillance and trauma exposure patterns that differ from those in traditional combat. By integrating recent advances in molecular biology, neuroimaging and clinical psychology, this review highlights the epidemiological characteristics, neurobiological mechanisms, clinical manifestations and treatment challenges of PTSD in both UAV operators and ground troops affected by UAV activities. By providing a comprehensive perspective on the mental health issues stemming from this novel mode of warfare, this article aims to inform the development of targeted prevention and intervention strategies to better support the psychological resilience of military personnel in the era of unmanned combat.

## INTRODUCTION

Unmanned aerial vehicles (UAVs), commonly referred to as drones, have become integral to modern military operations, fundamentally transforming the nature of warfare. Initially deployed mainly for intelligence, surveillance and reconnaissance (ISR) missions, drones have evolved to undertake direct combat roles, including precision strikes and targeted eliminations. This evolution has enabled militaries to conduct ‘remote warfare’, allowing operators to engage adversaries from geographically distant control stations, thereby reducing immediate physical risks to personnel and potentially lowering operational costs. The strategic advantages of drones encompass persistent battlefield presence, rapid information gathering and the capability to execute precise attacks with minimal collateral damage, collectively shaping the dynamics of conflicts without necessarily achieving immediate territorial gains.^1^ The ongoing conflict in Ukraine exemplifies the tactical utility of drones, as both sides have leveraged UAVs to offset disparities in manpower and firepower, highlighting their force‐multiplying effect.^2^

However, the integration of drones into combat operations introduces novel psychological challenges, particularly for operators engaged in remote combat. Unlike traditional frontline soldiers, drone operators conduct prolonged surveillance and execute lethal actions via screens, often witnessing the real‐time consequences of their strikes while remaining physically detached from the battlefield. This unique environment can engender distinct forms of psychological stress, including emotional disengagement, moral injury and a sense of detachment, sometimes referred to as ‘screen trauma’.^1^, ^2^ Empirical studies have documented elevated risks of emotional exhaustion, burnout and post‐traumatic stress disorder (PTSD) symptoms among operators and support staff.^1^ Conversely, some research, including a longitudinal study of U.S. Army enlisted drone operators, suggests a lower incidence of PTSD than that among traditional combat troops, a difference possibly attributed to variations in trauma exposure, although the underlying mechanisms remain unclear.^3^

Concurrently, ground forces face evolving trauma exposures linked to drone warfare. The persistent aerial surveillance and the imminent threat of precise strikes introduce new dimensions of psychological stress for soldiers on the ground. The unpredictability and omnipresence of drone threats can exacerbate feelings of vulnerability and hypervigilance, contributing to the development of PTSD and related disorders.^4^ The psychological burden extends beyond combatants to affected civilians in conflict zones, who may experience chronic stress and trauma due to the constant presence of drones and the fear of strikes.^2^ This broad spectrum of exposure necessitates a comprehensive understanding of the mechanisms underpinning PTSD in the context of drone warfare.

PTSD, first formally recognised in 1980, is characterised by intrusive memories, avoidance behaviours, negative alterations in cognition and mood, and heightened arousal after exposure to traumatic events.^5^ Its diagnosis and management have evolved to encompass complex presentations, such as complex PTSD (CPTSD), which involves additional disturbances in self‐organisation, including affective dysregulation, negative self‐concept and interpersonal difficulties.^6^, ^7^ Neurobiological research has identified alterations in brain structures (e.g., the entorhinal cortex and anterior cingulate cortex (ACC)), functional connectivity and autonomic nervous system reactivity as correlates of PTSD severity, providing objective biomarkers that may aid in diagnosis and treatment stratification.^8^, ^9^ Psychological factors such as trauma‐related shame, guilt and negative cognitive appraisals have been implicated in symptom persistence, underscoring the importance of addressing these dimensions in therapy.^10^, ^11^

Social support and psychological resilience are critical protective factors that mitigate PTSD risk across diverse populations, including military personnel.^12^, ^13^ The mediating role of psychological security and the buffering effects of social networks underscore the necessity of integrating psychosocial interventions with clinical treatments to enhance recovery.^12^, ^14^ Furthermore, the interplay between PTSD and comorbid conditions such as depression, anxiety and burnout complicates clinical presentations, necessitating comprehensive assessment and tailored treatment approaches.^11^, ^15^

In the military context, longitudinal studies have delineated heterogeneous trajectories of PTSD symptoms, ranging from resilience to chronic and delayed‐onset patterns. These trajectories are influenced by factors including age, deployment length, trauma history, sleep disturbances and occupational stressors.^16^, ^17^, ^18^ Emerging evidence further suggests that early‐life stress (ELS) serves as a potent vulnerability factor, modulating neurobiological systems and significantly increasing susceptibility to PTSD after adult trauma exposure.^19^ These findings underscore the complexity of PTSD development and highlight the necessity for proactive monitoring and early intervention strategies within military populations. Furthermore, the evolving nature of warfare, characterised by multidomain operations and contested evacuation scenarios, necessitates adaptive behavioural health frameworks capable of providing layered, in‐theatre care to sustain combat effectiveness and psychological readiness.^20^

The psychological impact of drone warfare extends beyond operators to targeted individuals and communities. Emerging evidence from conflict zones, such as Ukraine, indicates an elevated prevalence of PTSD among civilians and military personnel exposed to drone attacks. Furthermore, parental PTSD symptoms correlate with an increased risk in children, highlighting the intergenerational transmission of trauma.^21^ The ethical and psychological dimensions of drone warfare necessitate further research to elucidate the full spectrum of mental health consequences and to develop culturally sensitive, scalable interventions.

In summary, the integration of drones into modern warfare has introduced complex psychological challenges for both operators and those exposed to drone‐related trauma. Understanding the unique psychosocial and biological mechanisms of PTSD in this context is essential for developing effective prevention, diagnosis and treatment strategies. This review aims to synthesise current knowledge on the dual perspectives of drone operators and targeted ground forces, analyse underlying mechanisms and discuss emerging therapeutic approaches to address the mental health needs arising from drone warfare.

## THE EVOLUTION OF DRONE BATTLEFIELD APPLICATIONS AND THEIR PSYCHOLOGICAL ENVIRONMENT

### From support to strike: The expansion of UAV roles and the evolution of psychological burden

The evolution of UAVs in modern warfare has significantly transformed the roles and psychological demands placed on their operators. Initially, UAVs were primarily employed for ISR missions, where operators were tasked with gathering and processing vast amounts of battlefield information. In this early phase, psychological stress was predominantly linked to the cognitive load and fatigue associated with prolonged, intense concentration. The necessity to maintain vigilance over extended shifts, often involving monotonous screen monitoring, imposed a sustained cognitive burden that could lead to mental exhaustion. This cognitive strain, although substantial, was largely confined to information processing and did not directly involve lethal decision‐making.

However, the advent of ‘hunter‐killer’ or strike‐capable UAVs has expanded the operational scope by integrating reconnaissance with direct engagement. This technological progression has transformed the operator’s role from a passive observer to an active participant in the kill chain, fundamentally altering the nature of their psychological burden. Operators are now tasked with making real‐time lethal decisions, introducing complex moral and emotional challenges. Consequently, the cognitive load now includes not only information processing but also the ethical implications of life‐and‐death decisions. This transition exposes operators to unique stressors, including the responsibility for targeting decisions and the consequences of strikes, which may involve collateral damage.

A particularly distressing aspect is the phenomenon often described as the ‘executioner’s perspective’, wherein operators repeatedly review footage of their strikes. This repeated exposure to the aftermath of their actions can engender profound moral injury and emotional distress, as operators grapple with the human cost of their decisions.^22^, ^23^ Concerns about unintended civilian harm further exacerbate this psychological strain, fostering anxiety and moral conflict. Additionally, the pressure to make timely decisions in dynamic combat environments can lead to decision paralysis or delayed responses, contributing to heightened stress and feelings of inadequacy. Collectively, these factors illustrate how the evolution of UAV roles from support to strike has introduced a multifaceted psychological burden encompassing cognitive, moral and emotional dimensions.

### The unique psychological environment of remote warfare: Alienation, moral dilemmas and sustained vigilance

Despite their physical separation from the battlefield, UAV operators encounter a unique psychological environment marked by alienation, moral conflict and chronic stress. Physical distance does not imply psychological detachment; instead, it may amplify isolation due to the lack of direct sensory input and the absence of camaraderie typical among deployed troops. This detachment can intensify loneliness, depriving operators of the social buffers that alleviate stress in traditional combat settings. Furthermore, the lack of immediate peer support contributes to increased vulnerability to psychological distress.

A central challenge is the moral dilemma inherent in conducting lethal operations from a secure, remote environment. This ‘clean killing’ paradigm, wherein operators execute deadly force without physical risk, often conflicts with internal moral frameworks. The dissonance between the clinical detachment required for strikes and the innate human aversion to taking life can precipitate moral injury, a condition closely linked to PTSD. Moral injury arises from the violation of deeply held ethical beliefs and can manifest as guilt, shame and profound emotional turmoil.^22^, ^24^, ^25^ This conflict is compounded by the operators’ awareness of potential civilian casualties, which may haunt them long after missions conclude.

Operational demands further contribute to psychological vulnerability. Typically working 12‐h shifts sustained over weeks or months, operators engage in continuous surveillance and strike readiness. This prolonged high‐stakes monitoring fosters chronic stress and sleep deprivation, both well‐established risk factors for PTSD. Sleep disturbances impair cognitive function and emotional regulation, diminishing resilience and increasing susceptibility to trauma‐related symptoms. Furthermore, the relentless vigilance required imposes a sustained state of hyperarousal, which can lead to burnout and psychological exhaustion.

Empirical studies have underscored the prevalence of PTSD and related conditions among UAV operators, highlighting the interplay of isolation, moral injury and chronic stress.^22^, ^24^, ^25^ The unique psychological environment of remote warfare necessitates comprehensive support systems addressing not only cognitive demands but also moral and emotional challenges. Recognising the distinct nature of these psychological burdens is critical for developing effective prevention and treatment approaches for this specialised military cohort.

## EPIDEMIOLOGY AND CLINICAL MANIFESTATIONS OF UAV‐RELATED PTSD IN MILITARY PERSONNEL

### PTSD prevalence and symptomatology among drone operators

Despite the absence of direct, traditional battlefield exposure to life‐threatening events, drone operators exhibit a prevalence of PTSD comparable to, and in some cases exceeding, that of certain combat troops. This finding challenges conventional PTSD diagnostic criteria, which emphasise direct exposure to trauma. Studies document that drone operators frequently experience intrusive symptoms, such as recurrent distressing memories and vivid flashbacks of strike footage, which can be as impactful as direct combat exposure.^1^, ^26^ Emotional numbing and hypervigilance are also prominent, reflecting persistent psychological arousal. Moreover, operators often endure significant moral injury and survivor guilt stemming from the ethical complexities of remote killing and awareness of collateral damage, intensifying their psychological burden beyond typical PTSD symptom clusters. The clinical presentation is further complicated by high rates of comorbid conditions, including depression, anxiety and substance use disorders, creating a multifaceted symptom profile that complicates diagnosis and treatment.^1^, ^26^

Specific prevalence data are emerging but remain heterogeneous. Nelson and colleagues’ large‐scale study of U.S. Army drone operators found a PTSD incidence of 8.2% within 24 months (95% confidence interval not reported), which is lower than the rates among deployed combat troops (typically 10%–18%) but still clinically significant.^3^ This finding must be interpreted cautiously: The lower rates may reflect diagnostic exclusion (operators not meeting Criterion A for direct threat exposure), underreporting due to stigma or genuine protective effects. The latter possibility warrants balanced consideration—remote operations may indeed buffer certain forms of direct combat trauma, such as witnessing dismemberment or experiencing life‐threatening personal danger. However, the qualitative severity of UAV‐related psychological distress (e.g., moral rumination and emotional exhaustion) may be comparable to, or even exceed, that of traditional PTSD in specific domains.^2^, ^3^ This constellation of symptoms underscores the need for a nuanced understanding of PTSD among drone operators, acknowledging that indirect trauma exposure can result in profound psychological consequences while also recognising that remote operations may spare operators from some of the most visceral horrors of close‐quarters combat.

Furthermore, not all UAV operators experience the same psychological burden. A growing body of evidence suggests that role‐specific differences are critical: Operators engaged solely in ISR missions report lower rates of moral distress and intrusive symptoms than those involved in ‘hunter‐killer’ strike missions. The latter group, who directly initiate lethal actions, exhibit significantly higher levels of guilt, shame and post‐mission rumination.^2^, ^3^ This granular, role‐based analysis is essential for understanding differential risk and tailoring preventive interventions.

### PTSD risk among ground forces exposed to drone threats

For ground forces, drone warfare presents a novel and unpredictable source of trauma that significantly increases PTSD risk. Unlike traditional combat threats, drones maintain a constant presence, combining continuous surveillance with the ability to execute sudden, precise strikes from above. This omnipresent threat environment creates a sustained state of hypervigilance and helplessness among soldiers, as the invisible and often untraceable nature of drone attacks undermines their sense of safety and control. Such exposure patterns closely align with the symptomatology of CPTSD, which extends beyond core PTSD symptoms to include difficulties in emotional regulation, a persistent negative self‐concept and impaired interpersonal relationships. Recent research on Ukrainian armed forces engaged in ongoing conflict observed a notable CPTSD prevalence of 21.5% among combat‐exposed personnel.^2^, ^20^

However, the evidence regarding the psychological impact on targeted individuals and communities remains limited. Most studies focus on UAV operators rather than on ground forces, civilians or entire communities living under constant drone surveillance. The ‘other side’ of drone warfare—those who are targeted or who live in fear of being targeted—has received far less empirical attention. Available qualitative reports suggest that affected populations experience chronic helplessness, a pervasive fear of the sky (‘lithophobia’) and collective trauma that may be transmitted across generations.^2^, ^21^ This review explicitly acknowledges this gap and calls for future research to systematically investigate the psychological consequences for ground forces and civilians exposed to drone operations, including the development of validated assessment tools for drone‐related community trauma.

### Role‐specific differences: ISR‐only versus hunter‐killer operators

As noted above, the psychological profile of UAV operators varies substantially by operational role. Operators restricted to ISR missions experience vicarious trauma through prolonged observation of distressing events (e.g., deaths, injuries and destruction) without directly causing harm.^3^ Their primary stressors include emotional exhaustion from continuous exposure to suffering and a sense of helplessness when unable to intervene. In contrast, hunter‐killer operators—who select targets and execute strikes—face additional burdens of direct moral agency, including guilt over killing, anxiety about collateral damage and post‐mission review of strike footage.^2^, ^22^ Preliminary evidence suggests that hunter‐killer operators have higher rates of subclinical moral distress and burnout (as measured by the Maslach Burnout Inventory) than ISR‐only operators, though formal PTSD diagnostic rates may not differ significantly.^3^ This role‐based distinction has important implications for screening and intervention design.

## COMPARATIVE MECHANISMS: TRADITIONAL PTSD VERSUS UAV‐RELATED PTSD

The integration of UAVs into modern warfare has precipitated a paradigm shift in the nature of combat‐related psychological trauma, necessitating a nuanced understanding of how PTSD manifests differently in drone operators compared to traditional combat personnel. Although both groups may exhibit overlapping symptomatology, the underlying aetiological pathways, neurobiological correlates and phenomenological presentations differ substantially. This section systematically compares the mechanisms of traditional PTSD—rooted in direct physical threat and sensory immersion—with UAV‐related PTSD, which is characterised by moral injury, existential crisis and the unique psychological burdens of remote warfare.^1^, ^26^, ^27^ Table 1 summarises these key differences.

**TABLE 1:** Comparison of traditional combat PTSD and UAV‐related PTSD

Feature	Traditional combat PTSD	UAV‐related PTSD
Primary trauma mechanism	Direct threat to self (fear conditioning)	Moral injury and perpetration‐induced stress
DSM‐5 Criterion A	Typically met (life threat)	Often not met (remote killing)
Core neurobiological correlates	Amygdala hyperreactivity, HPA axis dysregulation	DMN hyperactivation, altered moral cognition networks
Hypervigilance orientation	External (environmental threats)	Internal (moral rumination, guilt)
Traumatic memory quality	Sensory‐rich fragments	Abstract, narrative‐based, morally elaborated
Common comorbidity	Substance use, externalising disorders	Burnout, emotional exhaustion, adjustment disorders
Key protective factor	Unit cohesion, shared experience	Social isolation

Abbreviations: DMN, default mode network; DSM‐5, Diagnostic and Statistical Manual of Mental Disorders, Fifth Edition; HPA, hypothalamic–pituitary–adrenal; PTSD, post‐traumatic stress disorder; UAV, unmanned aerial vehicle.

### Differential pathophysiology: Threat‐based versus morally based trauma

Traditional PTSD, as defined by the Diagnostic and Statistical Manual of Mental Disorders (DSM‐5), requires exposure to actual or threatened death, serious injury or sexual violence. The pathophysiology of conventional combat‐related PTSD is predominantly driven by activation of the hypothalamic–pituitary–adrenal (HPA) axis and sympathetic nervous system in response to life‐threatening danger.^4^, ^8^ Neurobiologically, this manifests as heightened amygdala reactivity, impaired prefrontal cortical inhibition of limbic structures and altered fear‐extinction circuitry mediated by the hippocampus and the ventromedial prefrontal cortex.^28^, ^29^ The traumatic memory in traditional PTSD is encoded under extreme physiological arousal, leading to fragmented, sensory‐based recollections that intrude involuntarily.^30^

In contrast, UAV operators experience trauma through a fundamentally different mechanism: They are exposed to the lethal consequences of their actions without experiencing direct physical threat to themselves.^1^, ^2^ This ‘remote trauma’ paradigm challenges conventional PTSD diagnostic frameworks, as operators often do not meet the DSM‐5 Criterion A requirement of direct exposure to threatened death or serious injury.^2^, ^5^ Hernando Ortega’s extensive research on UAV operators found that none met full PTSD diagnostic criteria when DSM definitions were strictly applied, despite reporting significant psychological distress.^2^ Instead, operators exhibited what Ortega termed ‘existential conflict’—a form of distress characterised by post‐mission questioning, guilt about decision‐making and moral rumination that differs qualitatively from the fear‐based symptomatology of traditional PTSD.^2^

The concept of perpetration‐induced traumatic stress (PITS), proposed by Rachel MacNair, offers a more appropriate framework for understanding UAV‐related trauma.^2^ PITS specifically addresses psychological distress arising from being the agent of harm rather than the victim of threat. Unlike traditional PTSD, in which the individual experiences fear for their own safety, PITS involves guilt, shame and moral conflict stemming from one’s own actions that violate internal ethical standards. UAV operators represent a ‘pure’ form of perpetration‐induced trauma, as they engage in lethal action while being completely insulated from physical counter‐risk.^2^ This distinction is critical: Traditional PTSD is fundamentally a disorder of fear conditioning and threat processing, whereas UAV‐related psychological distress is primarily a disorder of moral cognition and self‐perception.

### Moral injury and existential crisis as core mechanisms

The concept of moral injury has emerged as a central framework for understanding UAV operator psychopathology, distinguishing it from traditional combat‐related PTSD.^22^, ^24^, ^27^ Moral injury refers to the psychological distress that results from actions, or the failure to act, which violate one’s deeply held moral or ethical beliefs. Nash and Litz conceptualise moral injury as distinct from fear‐based trauma, emphasising that when individuals engage in behaviour contradicting their personal moral standards—even under legitimate military authority—psychological disorder may follow.^22^, ^27^

For UAV operators, moral injury is compounded by several unique factors. First, the ‘clean killing’ paradigm creates profound dissonance between the clinical detachment required for remote operations and the innate human aversion to taking life.^2^, ^27^ Second, operators often conduct prolonged surveillance of targets, observing their daily routines and family interactions before executing strikes—a phenomenon that can foster psychological connection with targets and amplify post‐strike guilt.^6^, ^22^ Third, the aftermath of strikes is frequently reviewed in high‐definition footage, exposing operators to graphic imagery of death and destruction that they themselves caused.^6^, ^13^ As one operator described, the experience differs fundamentally from traditional combat: ‘We’re not playing a video game here’.^2^

Paul Bartone offers an alternative but complementary model, suggesting that combat may erode the sense of meaning in life, leading to a form of existential neurosis.^27^ This perspective is particularly relevant to UAV operators, who, although not personally experiencing trauma, witness or imagine the mortal consequences of their actions. Bartone’s framework posits that existential crisis may be accompanied by changes in cognition, affect and behaviour that overlap with PTSD symptoms—including emotional numbing, social withdrawal and dysphoria—but arise from meaninglessness and moral confusion rather than fear conditioning.^27^

Psychiatrist Larry Dewey’s clinical work with veterans further emphasises that the common denominator across combat‐related psychological disorders is the impact of killing on meaning‐making.^27^ Dewey maintains that how one kills matters less than the comprehension that one has taken the lives of others. This insight is critical for distinguishing UAV‐related trauma from traditional PTSD: Both involve killing, but traditional combatants kill within a context of mutual threat and self‐preservation, whereas UAV operators kill from a position of complete safety, potentially intensifying moral self‐scrutiny.^27^

### Distinct symptom profiles and comorbidity patterns

The clinical presentation of UAV‐related psychological distress differs from traditional PTSD in several important respects. Although both conditions may include sleep disturbances, hypervigilance and emotional numbing, the qualitative nature and aetiological drivers of these symptoms diverge.^2^, ^6^

In traditional combat PTSD, hypervigilance is typically oriented towards external threats—scanning environments for danger, startle responses to sudden noises and persistent concerns about physical safety.^8^, ^30^ In contrast, UAV operators’ hypervigilance is more internally directed, characterised by obsessive rumination about past decisions, moral self‐scrutiny and anxiety about the consequences of their actions.^2^, ^6^ Sleep disturbances in operators are frequently linked to guilt‐related dreams or intrusive thoughts about strike footage rather than combat re‐experiencing.^6^

Emotional numbing and detachment in traditional PTSD often serve as protective mechanisms against overwhelming fear and threat‐related arousal.^28^ In UAV operators, detachment may instead reflect moral disengagement strategies or the cumulative effect of ‘screen‐based’ emotional suppression required for operational effectiveness.^6^, ^27^ The phenomenon of ‘emotional whiplash’—the rapid transition between high‐stakes combat monitoring and domestic family life—creates a unique form of emotional compartmentalisation not experienced by deployed traditional forces.^6^, ^8^

Comorbidity patterns also differ. UAV operators demonstrate higher rates of adjustment disorders, relationship problems and subthreshold distress, whereas traditional combat veterans show more pronounced rates of full‐syndrome PTSD, substance use disorders and externalising behaviours.^3^, ^6^ As noted in the section: PTSD prevalence and symptomatology among drone operators, Nelson et al. reported an 8.2% 24‐month PTSD incidence among U.S. Army drone operators, which is lower than the rates among deployed combat troops (10%–18%).^3^ However, operators showed significant rates of emotional exhaustion, burnout and cynicism as measured by the Maslach Burnout Inventory.^6^, ^21^ This suggests that UAV‐related psychopathology may manifest more prominently as occupational burnout and moral distress rather than classic fear‐based PTSD.

### Epidemiological evidence supporting mechanistic distinction

Epidemiological studies provide empirical support for distinguishing UAV‐related trauma from traditional combat PTSD. Nelson and colleagues’ large‐scale study of U.S. Army drone operators found a lower PTSD incidence than that among traditional combat troops, but this finding must be interpreted cautiously.^3^ The lower rates may reflect diagnostic exclusion (operators not meeting Criterion A), underreporting due to stigma or genuine differences in trauma exposure patterns.^3^, ^6^

Importantly, studies examining intelligence coordinators separately, using the Vicarious Combat Exposure Scale, revealed that 12.9% reported high combat exposure and 39% reported moderate exposure, despite never having been physically present on the battlefield.^6^, ^22^ This vicarious trauma—witnessing combat consequences through screens—represents a distinct exposure pathway not captured by traditional PTSD frameworks. Rotating shift work, prolonged working hours (> 50 hours per week) and tenure exceeding 24 months were more strongly associated with psychological distress in operators than combat exposure variables.^6^, ^22^

A scientometric analysis of PTSD research by Sabé and colleagues identified a significant shift in research trends: ‘Research on war‐related trauma has shifted from battlefield‐related in‐person exposure trauma to drone operator trauma’.^1^, ^26^ This recognition reflects the growing acknowledgement that UAV‐related psychological distress represents a distinct clinical entity requiring separate conceptual frameworks and intervention approaches.

### The cyborg soldier and digital moral trauma: A new conceptual framework

The previous sections have established that UAV‐related psychological distress differs fundamentally from traditional combat PTSD, with moral injury and existential crisis serving as core mechanisms. To further elucidate how the unique human–machine interface of drone warfare shapes these psychological outcomes, we introduce two interconnected theoretical constructs: the cyborg soldier and digital moral trauma (DMT). These frameworks integrate insights from cognitive neuroscience, media theory and military ethics, offering a novel lens for understanding the neurocognitive underpinnings of trauma in remote warfare.

#### The cyborg soldier: Human–machine integration and its psychological consequences

The concept of the ‘cyborg’—a cybernetic organism blending human and machine—was originally popularised by Donna Haraway to critique traditional boundaries in identity and technology.^31^ In the military context, the cyborg soldier represents the deep integration of human operators with weapon systems, where the technological interface becomes an extension of the soldier’s perceptual and motor apparatus.^32^ UAV operators epitomise this fusion: They perceive the battlefield through cameras and sensors, act through remote manipulation mechanisms and experience combat consequences via screens and data streams. This technological embodiment fundamentally alters the phenomenological experience of warfare.

Drawing on Vanžura’s ‘extended mind’ theory, the UAV operator’s cognition is distributed across the human‐technology system.^2^ The drone’s sensors become the operator’s eyes; its weapons become their hands. This extension creates a paradoxical situation: Although the operator’s physical body remains safe in a control station hundreds or thousands of kilometres away, their cognitive and emotional systems are continuously immersed in the lethal realities of the battlefield. This remote presence blurs the boundaries between self and machine, combatant and civilian, perpetrator and witness.

Several psychological consequences arise from this cyborg state:

Identity diffusion: Operators may struggle to reconcile their dual identities as both peaceful family members at home and lethal combatants on the screen. This fragmentation can lead to what Grossman terms ‘emotional whiplash’—the rapid oscillation between high‐stakes combat monitoring and domestic life—which impairs emotional integration and self‐coherence.^6^, ^33^

Moral agency distortion: When lethal actions are mediated through technology, the sense of personal agency and responsibility may be diluted. Operators may experience a form of moral disengagement, in which the technological interface creates psychological distance from the consequences of their actions, potentially reducing the immediate emotional impact but paradoxically intensifying delayed moral rumination.^34^

Affective flattening: The screen‐based nature of remote warfare limits the rich sensory and emotional feedback inherent in face‐to‐face combat (e.g., the smell of blood, the sound of screams and the tactile experience of taking a life). This sensory deprivation may lead to a form of emotional numbing specific to the cyborg condition, in which operators learn to suppress affective responses to maintain operational effectiveness, only to have these emotions resurface later in distorted forms.^35^

#### Digital moral trauma: A new category of trauma

Building on the cyborg soldier framework, we propose the concept of DMT to capture the unique form of moral trauma engendered by technologically mediated warfare. DMT is defined as the psychological distress that arises when an individual, through a human–machine interface, engages in actions that violate their moral beliefs, yet the mediated nature of these actions creates distinct phenomenological and neurobiological features compared to traditional moral injury. Table 2 compares DMT with traditional moral injury.

**TABLE 2:** Comparison of traditional moral injury and DMT

Feature	Traditional moral injury	DMT
Mode of action	Direct, physical (e.g., close combat killing)	Mediated through technology (screen‐based)
Sensory feedback	Immediate, multimodal (sight, sound, smell, touch)	Delayed, restricted (primarily visual)
Temporal relationship	Action and consequence co‐occur	Often delayed (review of recorded footage)
Perpetrator‐victim distance	Proximal (face‐to‐face)	Distal (anonymous, remote)
Social context	Usually shared with unit/peers	Often isolated, limited debriefing
Neural correlates	Amygdala, ACC, insula (emotional/salience networks)	DMN, mPFC, TPJ (moral/self‐referential networks)
Core emotional theme	Shame, guilt, betrayal	Moral dissonance, existential questioning, ‘screen guilt’

Abbreviations: ACC, anterior cingulate cortex; DMN, default mode network; DMT, digital moral trauma; mPFC, medial prefrontal cortex; TPJ, temporoparietal junction.

Digital moral trauma differs from conventional moral injury in several key dimensions:

Mediation of action: In traditional moral injury, the individual directly commits or witnesses morally transgressive acts (e.g., killing a civilian in close combat). In DMT, the act is performed through a technological interface, introducing a layer of abstraction that can both buffer and intensify moral conflict. The operator may feel simultaneously responsible (as the agent who initiated the strike) and detached (as the button‐pusher behind a screen), creating a moral dissonance that is difficult to resolve.^36^

Temporal delay: Traditional moral injury typically involves immediate sensory feedback that anchors the memory in time and space. In DMT, the consequences of one’s actions are often witnessed later through recorded footage, creating a temporal dissociation between action and outcome. This delay can fragment the traumatic memory, making it more susceptible to intrusive replay and distorted rumination.^6^

Perpetrator‐victim distance: The physical separation between operator and target, combined with the anonymity of the screen, may reduce immediate empathy and increase moral disengagement. However, this same distance can later amplify guilt, as operators imagine the humanity of those they killed without the contextual cues that might facilitate acceptance.^37^

Lack of shared experience: Traditional combatants often process moral trauma within a community of peers who shared the same experiences. Drone operators, in contrast, frequently work in isolation, with limited opportunities to debrief or share the moral weight of their decisions. This social isolation compounds the moral trauma, preventing the communal sense‐making that can mitigate guilt.^38^

#### Neurocognitive distinctions between physical and virtual trauma

The digital nature of DMT implies that its neural encoding differs from that of physical trauma. Although traditional physical trauma activates threat‐processing circuits (amygdala, periaqueductal grey and hypothalamus) and fear‐conditioning pathways, virtual trauma engages a distinct network involving moral cognition, self‐referential processing and mental simulation.

Neuroimaging studies have shown that making moral decisions in virtual environments recruits the medial prefrontal cortex (mPFC), temporoparietal junction (TPJ) and precuneus—regions central to theory of mind and self‐reflection.^39^ When operators review strike footage, they are not merely recalling an event; they are actively simulating the experience of the target, imagining the human cost of their actions. This mental simulation engages the default mode network (DMN), particularly the posterior cingulate cortex and mPFC, which are involved in self‐referential thought and moral evaluation.^40^

The DMN’s role in DMT is crucial. As discussed in the section: Unique neurocognitive features of UAV operators: sustained surveillance and default mode network hyperactivation, sustained surveillance tasks lead to DMN hyperactivation in operators. This heightened self‐referential activity may predispose them to excessive moral rumination, as the brain remains in a state of self‐focused evaluation even when not engaged in combat tasks. Unlike physical trauma, where hyperarousal is mediated by sympathetic activation and the salience network, DMT may be characterised by DMN‐driven intrusive self‐reflection—a relentless, guilt‐laden replay of one’s actions from the perspective of both self and others.

Furthermore, the lack of direct sensory feedback in virtual trauma may lead to altered encoding in sensory cortices. Traditional traumatic memories often involve vivid sensory fragments (visual, auditory and olfactory) that trigger flashbacks. In DMT, the traumatic memory may be more abstract, cognitive and narrative‐based, involving less sensory richness but greater moral elaboration. This difference has implications for therapy: exposure‐based treatments that rely on sensory reactivation may be less effective for DMT, whereas cognitive and narrative approaches targeting moral appraisals may be more appropriate.^41^

#### Implications for research and clinical practice

The cyborg soldier and DMT frameworks challenge the field to move beyond traditional PTSD paradigms and develop assessment tools and interventions tailored to the unique neurocognitive profile of remote warfare trauma. Future research should employ virtual reality paradigms to experimentally manipulate moral distance and technological mediation, examining their effects on neural activity and psychological outcomes. Neuroimaging studies should focus on DMN connectivity and moral cognition networks in drone operators, comparing them with those of traditional combat veterans. Clinically, interventions must address the specific phenomenology of DMT, incorporating strategies to reduce moral disengagement, facilitate ethical integration and promote social connection among isolated operators.

## NEUROBIOLOGICAL AND EPIGENETIC MECHANISMS OF UAV‐RELATED PTSD

### Neuroimaging findings: The role of prefrontal–limbic circuitry and the hippocampus

Neuroimaging studies have significantly advanced our understanding of the neural substrates underlying PTSD in soldiers, emphasising critical alterations in prefrontal–limbic circuitry and the hippocampus. Soldiers with PTSD often display volumetric reductions and cortical thinning in prefrontal regions, such as the superior and middle frontal gyri, which are essential for emotional regulation and cognitive control.^28^, ^42^ These structural deficits align with functional imaging findings revealing hypoactivation of the mPFC and dorsolateral prefrontal cortex (DLPFC), both involved in the top‐down modulation of limbic structures, including the amygdala. This reduced prefrontal control is believed to contribute to hallmark PTSD symptoms, such as heightened emotional reactivity and impaired fear extinction.

The hippocampus, crucial for memory consolidation and contextual processing, exhibits significant abnormalities in patients with PTSD. Prospective studies indicate that hippocampal volume asymmetry, particularly a larger right hippocampus, can predict PTSD symptom onset after deployment, suggesting that hippocampal morphology may serve as a vulnerability biomarker.^29^ Furthermore, functional connectivity analyses reveal disrupted communication between the hippocampus and prefrontal regions, undermining the integration of contextual information essential for effective fear extinction and emotional regulation.^30^ This disruption may be particularly pronounced in drone operators facing chronic cognitive demands requiring sustained attention, moral decision‐making and emotional regulation, potentially leading to maladaptive neural plasticity within these circuits.

Neuroimaging evidence also indicates altered activation patterns in the ACC and the bed nucleus of the stria terminalis (BNST), both crucial regions involved in stress response and emotional processing.^43^ Patients with PTSD exhibit increased BNST activation and demonstrate altered functional connectivity with the prefrontal cortex and limbic structures. These alterations correlate with symptom severity and HPA axis dysfunction.^43^ Collectively, these findings underscore a model of dysexecutive emotion processing in PTSD, wherein impaired prefrontal–limbic interactions contribute to traumatic stress symptom persistence.

### Epigenetics and molecular features: From susceptibility to disease markers

Epigenetic research has elucidated the molecular foundations of PTSD, demonstrating how environmental stressors, such as combat exposure and drone operation, can induce enduring gene expression modifications without altering the DNA sequence. In military personnel, epigenetic changes, particularly DNA methylation alterations in genes associated with synaptic plasticity and stress response, have been correlated with PTSD susceptibility and symptomatology. For example, decreased methylation of the brain‐derived neurotrophic factor (*BDNF*) gene, which plays a critical role in synaptic plasticity and neural resilience, has been linked to PTSD diagnosis, suggesting compromised neuroplastic mechanisms.^44^ Likewise, hypomethylation of the *NR3C1* gene, encoding the glucocorticoid receptor involved in HPA axis regulation, correlates with PTSD, indicating dysregulation of stress hormone signalling pathways.^43^

Multiomic approaches integrating proteomic, metabolomic and epigenomic data have identified consistent molecular signatures in soldiers with PTSD, characterised by heightened inflammatory responses, oxidative stress and metabolic disturbances.^42^ These molecular alterations reflect the systemic impact of chronic stress and may contribute to PTSD pathophysiology by affecting neuronal function and plasticity. Notably, a longitudinal study of Burundian soldiers linked differential methylation in genes involved in linoleic acid metabolism to changes in PTSD symptoms over time, highlighting the dynamic nature of epigenetic regulation in response to trauma and its potential as a biomarker of disease progression.^45^

Critically, a recent review by Zhang and colleagues synthesises clinical evidence demonstrating that ELS triggers long‐lasting alterations in neural plasticity and HPA axis regulation, establishing ELS as a key risk factor for the later development of PTSD after trauma exposure in adulthood.^19^ The chronic cognitive and emotional demands placed on drone operators may exacerbate these epigenetic changes, as sustained stress exposure can modulate gene expression through epigenetic mechanisms, thereby influencing neural circuitry and behavioural outcomes. This suggests that the unique operational environment of drone warfare not only affects brain structure and function but also leaves molecular imprints that mediate vulnerability and resilience to PTSD. Understanding these epigenetic and molecular features offers promising avenues for developing targeted interventions and personalised treatments.

### Unique neurocognitive features of UAV operators: Sustained surveillance and default mode network hyperactivation

Beyond the neurobiological alterations common to traditional PTSD, UAV operators exhibit distinctive neurocognitive characteristics shaped by their unique operational environment. Among the most significant is DMN dysfunction, particularly hyperactivation resulting from prolonged sustained surveillance tasks and its aberrant coupling with task‐positive networks.

#### Default mode network function and its classical alterations in PTSD

The DMN is a set of brain regions—including the posterior cingulate cortex, medial prefrontal cortex, precuneus and angular gyri—that are highly active during rest, self‐referential thought, episodic memory retrieval and future planning. In healthy individuals, the DMN is typically suppressed during externally focused tasks, allowing cognitive resources to be allocated to current demands. This dynamic equilibrium between task‐positive networks and the DMN is fundamental to healthy cognitive function.

In traditional PTSD research, DMN abnormalities have been widely reported. Patients with PTSD often exhibit reduced within‐network connectivity, particularly decreased functional connectivity between the posterior cingulate cortex and hippocampus/parahippocampal regions, which may relate to impaired integration of traumatic memories and altered episodic memory retrieval.^40^ Additionally, patients with PTSD show blurred boundaries between the DMN, salience network and central executive network—reduced network segregation that may allow intrusive thoughts to infiltrate during rest or impair cognitive control during tasks.

#### Unique cognitive demands of UAV operators and associated DMN alterations

The neurocognitive profile of UAV operators differs fundamentally from that of patients with traditional PTSD, stemming from the distinct demands of their operational environment. A core feature of UAV warfare is sustained surveillance—operators must maintain prolonged vigilance, continuously monitoring screens for dynamic information, ready to identify threats and respond instantaneously.^2^, ^46^ This task demand contrasts sharply with traditional combat exposure patterns: Although traditional combatants experience intermittent, high‐intensity threat exposure, UAV operators face continuous, low‐intensity cognitive load sustained over hours, months and even years without interruption.

From a neuroimaging perspective, this sustained surveillance state may produce two critical forms of DMN abnormality:

First, task‐related DMN hyperactivation. In healthy individuals, the DMN should be suppressed during externally focused tasks. However, for operators engaged in long‐term sustained surveillance, the brain may develop a maladaptive neural strategy—the DMN fails to fully deactivate even when concentrated attention is required. This DMN disinhibition may allow operators to be intruded upon by self‐referential thoughts, moral reflections or traumatic imagery, even while performing critical monitoring tasks, creating cognitive interference.

Second, resting‐state DMN hyperactivation. Prolonged high‐intensity surveillance may elevate baseline DMN activity during rest periods. This hyperactivation manifests as an inability to disengage from work‐related cognition even after leaving the operational environment—operators may engage in relentless mental rumination, repeatedly reviewing past decisions, imagining missed threats or worrying about the consequences of their actions.^2^, ^46^ ‘Combat partitioning’—the rapid switching between remote combat and domestic life—is a core stressor. This daily switching may further exacerbate DMN hyperactivation, as operators' brains struggle to transition from ‘battlefield mode’ to ‘home mode’.

#### Clinical significance of DMN hyperactivation and intervention implications

DMN hyperactivation is directly linked to specific clinical symptoms in UAV operators. First, it relates to intrusive symptoms—operators are more susceptible to task‐related intrusive imagery or morally distressing thoughts during rest or attempted sleep.^2^, ^46^ Second, it correlates with emotional exhaustion and burnout—a sustained cognitive load combined with an inability to effectively ‘shut down’ the DMN may lead to excessive neural resource depletion, manifesting as fatigue, reduced motivation and emotional numbness. Finally, it may exacerbate the neural basis of moral trauma—the DMN is involved in self‐referential processing and moral judgement, and its hyperactivation may intensify negative self‐evaluation of one's actions, creating a vicious cycle of ‘moral rumination’.^46^

From an intervention perspective, strategies targeting DMN hyperactivation may include mindfulness‐based interventions, training operators to recognise and release intrusive thoughts and reducing spontaneous DMN overactivity; cognitive rehabilitation training, enhancing dynamic switching between task‐positive and task‐negative networks; and optimised shift scheduling, providing adequate ‘neural offline’ time to restore normal DMN rhythmicity.

## COMORBID CONDITIONS AND COMPLEX CLINICAL PRESENTATIONS: PAIN, COGNITION AND DISSOCIATION

### Comorbidity of chronic pain, PTSD and opioid use

Chronic pain and PTSD frequently co‐occur among military personnel, creating a complex clinical picture that significantly impacts treatment outcomes and healthcare utilisation. Epidemiological data from large veteran populations indicate that approximately 21.6% of individuals with chronic pain also meet PTSD criteria, whereas, conversely, over half of those with PTSD have chronic pain diagnoses.^47^ This high comorbidity is associated with increased symptom severity, greater functional impairment and elevated psychiatric comorbidities compared to either condition alone. Veterans with both conditions are more likely to receive opioid prescriptions exceeding 30 days.^48^ Furthermore, PTSD appears to exacerbate pain intensity and psychological distress, leading to more complex pain experiences and poorer prognoses.^49^

Physiological studies provide insight into underlying mechanisms. Soldiers with coexisting chronic pain and PTSD exhibit heightened autonomic nervous system reactivity to negative emotional stimuli, as evidenced by increased low‐frequency to high‐frequency heart rate variability ratios, indicating sympathetic overactivation.^50^ This autonomic dysregulation may contribute to the maintenance and amplification of both pain and PTSD symptoms. Moreover, neurobiological models suggest shared pathways involving emotional processing and pain perception overlap, with avoidance behaviours in PTSD paralleling pain avoidance, thereby reinforcing symptom chronicity.^51^ The interplay between pain catastrophising and distress intolerance further mediates the relationship between PTSD symptom severity and pain outcomes, with the helplessness dimension of catastrophising being particularly influential.^52^

The management of this comorbidity is complicated by an increased risk of opioid misuse and dependence. PTSD symptom severity correlates with higher rates of opioid misuse, risky alcohol use and concurrent benzodiazepine prescriptions among individuals with chronic pain.^53^ Social determinants such as health literacy and emotional support also modulate opioid use patterns; for instance, lower emotional support intensifies the association between PTSD and persistent opioid use.^54^ These findings underscore the necessity for comprehensive assessment and tailored interventions addressing both pain and PTSD symptoms while mitigating opioid‐related risks.

Nonpharmacological interventions have demonstrated significant potential in reducing opioid reliance and enhancing outcomes. Multidisciplinary pain rehabilitation programmes incorporating cognitive behavioural therapy (CBT), yoga and acceptance and commitment therapy (ACT) have shown efficacy in alleviating pain and PTSD symptoms, reducing kinesiophobia and decreasing opioid and benzodiazepine use.^55^, ^56^, ^57^ Notably, soldiers with comorbid PTSD exhibit favourable responses to behavioural pain treatments, including yoga, with no evidence suggesting that PTSD undermines treatment effectiveness.^58^ Furthermore, integrating nonpharmacological modalities such as acupuncture and biofeedback has been linked to a reduced likelihood of prolonged opioid prescriptions among soldiers with chronic pain and PTSD.^48^

### Cognitive impairment and dissociative symptoms

Cognitive dysfunction and dissociative symptoms are prevalent and clinically significant features in soldiers with PTSD, particularly when comorbid with mild traumatic brain injury (mTBI) and chronic pain. Even subclinical depressive symptoms have been linked to diminished attentional performance, indicating that cognitive impairments may manifest early and subtly.^59^ The interaction between mTBI and PTSD symptoms differentially affects cognitive domains depending on age; younger soldiers may experience more pronounced frontal lobe‐related deficits, whereas older soldiers exhibit broader cognitive impairments.^59^ This heterogeneity complicates clinical assessment and necessitates age‐ and injury‐specific evaluation protocols.

Dissociation, a psychological defence mechanism characterised by disruptions in consciousness, memory and identity, is highly prevalent among combat‐exposed soldiers, with reported rates as high as 76.6% in some studies.^59^ Dissociative symptom severity correlates strongly with PTSD severity, suggesting that dissociation may exacerbate the clinical course of PTSD by interfering with trauma memory processing and emotional regulation. This interference can hinder the integration of traumatic memories, perpetuating symptomatology and complicating therapeutic interventions.^59^ Dissociation also poses diagnostic challenges, as it may mask or mimic other psychiatric or neurological conditions, thereby delaying appropriate treatment.

The coexistence of cognitive impairments and dissociative symptoms in soldiers with PTSD and chronic pain presents a complex clinical profile complicating both diagnosis and management. These overlapping symptom domains may contribute to increased functional disability, poorer treatment adherence and reduced responsiveness to conventional therapies. For instance, cognitive deficits can hinder effective engagement in cognitive behavioural interventions, whereas dissociative symptoms may restrict the emotional processing necessary for trauma‐focused therapies. Additionally, chronic pain can exacerbate cognitive dysfunction through mechanisms such as attentional interference and neuroinflammation.^59^

Given these complexities, integrated assessment strategies encompassing neuropsychological testing, dissociative symptom evaluation and pain assessment are critical for accurate diagnosis and personalised treatment planning. Emerging evidence supports multidisciplinary approaches concurrently addressing cognitive rehabilitation, trauma‐focused psychotherapy and pain management. Such approaches may include cognitive remediation therapies, mindfulness‐based interventions and pharmacological treatments targeting neurobiological substrates implicated in cognitive and dissociative symptoms.

## TREATMENT CHALLENGES, INTERVENTION STRATEGIES AND PREVENTION PROSPECTS

### Treatment engagement barriers and ‘intentional avoiders’

Low treatment engagement among soldiers with PTSD continues to pose a significant challenge in military mental healthcare. A notable subset, often referred to as ‘intentional avoiders’, recognises their psychological difficulties but intentionally abstains from seeking treatment due to negative perceptions of psychotherapy, heightened self‐reliance and stigma associated with mental illness. These avoidant behaviours align with hallmark PTSD symptomatology, where avoidance of trauma‐related stimuli acts as a maladaptive coping mechanism obstructing therapeutic engagement.^60^, ^61^, ^62^

In the context of UAV operators, treatment strategies must be customised to address their unique trauma profiles, particularly moral trauma and trauma memories associated with screen‐based combat exposure. Traditional exposure therapies, which depend on direct confrontation with trauma cues, may need adaptation to effectively engage operators whose traumatic experiences are mediated through remote visual stimuli rather than direct physical threats. Eye movement desensitisation and reprocessing (EMDR) therapy has shown efficacy within military populations, with treatment success correlating with improvements in sleep parameters such as rapid eye movement (REM) density. Importantly, the frequency of pretreatment sleep disruptions can predict the number of EMDR sessions required, highlighting the necessity of integrating sleep assessments into treatment planning.^60^, ^61^, ^62^ Addressing the psychological barriers faced by intentional avoiders requires interventions that not only diminish stigma and negative treatment beliefs but also incorporate modalities sensitive to the specific trauma experiences of drone operators.

### Advances in pharmacological and nonpharmacological interventions

Pharmacological interventions for PTSD have been studied extensively, though most trials have focused on traditional combat‐related PTSD rather than UAV‐specific presentations. Selective serotonin reuptake inhibitors (SSRIs), including sertraline and paroxetine, remain the first‐line pharmacotherapy for PTSD, with meta‐analyses showing modest effect sizes (Hedges' *g* ≈ 0.3–0.4).^63^ However, response rates are variable, and many patients remain symptomatic. Ketamine has yielded mixed results in military populations: Meta‐analyses indicate that ketamine does not significantly reduce PTSD incidence among combat‐exposed soldiers and may be ineffective or even detrimental in early PTSD stages. However, some evidence suggests symptomatic improvement in chronic PTSD cases.^63^

Emerging research has explored natural products with potential neuroprotective and anti‐inflammatory effects. Preclinical studies have identified tanshinone IIA (a diterpene quinone from *Salvia miltiorrhiza*) and evodiamine (an alkaloid from *Evodia rutaecarpa*) as candidate agents that may modulate stress responses and reduce PTSD‐like behaviours in animal models, possibly through regulation of the HPA axis, reduction of oxidative stress and enhancement of neuroplasticity.^64^, ^65^ Although these compounds have not yet been tested in clinical trials for PTSD, they represent promising future research directions, particularly for UAV‐related trauma, where inflammatory mechanisms may be relevant.^42^

Nonpharmacological interventions have shown promising potential, especially those targeting autonomic nervous system regulation through biofeedback and mindfulness‐based practices. Techniques such as heart rate variability biofeedback and mindfulness training can modulate physiological arousal and enhance sleep quality, which is critical given that elevated sleep heart rate during stress training predicts subsequent PTSD risk. Interventions addressing sleep disturbances, including improving sleep heart rate and REM sleep quality, are emerging as vital components of comprehensive PTSD management.^63^, ^64^, ^65^ Furthermore, novel treatment models emphasising self‐efficacy and directly addressing negative beliefs about therapy are essential for overcoming treatment avoidance among active‐duty personnel. These models aim to empower soldiers, reduce stigma and foster sustained engagement with evidence‐based treatments. For UAV‐specific populations, future research should investigate whether combination approaches—such as SSRIs plus cognitive behavioural therapy tailored to address digital moral trauma—yield superior outcomes.

## FUTURE RESEARCH DIRECTIONS AND INTEGRATED MANAGEMENT FRAMEWORK

### Construction of precision prevention and risk assessment models

The development of precision prevention and risk assessment models for PTSD among military personnel, particularly UAV operators, relies on leveraging predeployment data to identify individuals at heightened vulnerability to combat‐related stressors. By utilising comprehensive datasets encompassing psychological scales, physiological indicators and emerging biomarkers such as epigenetic modifications, researchers can construct predictive models stratifying soldiers based on PTSD susceptibility. For instance, a large‐scale retrospective cohort study of Israel Defense Forces conscripts demonstrated that preservice assessments of adverse childhood experiences, including sexual abuse and emotional neglect, were significantly associated with an increased risk of combat‐related PTSD, underscoring the importance of early psychological profiling in risk stratification.^66^ Similarly, predictive scoring systems incorporating variables such as prior psychiatric history and trauma exposure have effectively differentiated soldiers exhibiting varying degrees of psychological resilience to combat stressors. These models facilitate targeted preventive interventions by identifying high‐risk individuals prior to deployment, thereby optimising resource allocation and enhancing mental health support.

Future advancements could integrate UAV operator‐specific psychological assessments, including measures of moral sensitivity and empathy, which are critical given the unique ethical and emotional challenges faced by drone operators. Incorporating such domain‐specific psychological traits into risk models may enhance predictive accuracy and enable tailored resilience training. Moreover, incorporating neurobiological and epigenetic markers, as suggested by recent findings on microRNA dysregulation associated with PTSD symptom trajectories in military cohorts, could provide mechanistic insights and refine risk stratification.^67^ Integrating multidimensional data—psychological, physiological and molecular—into sophisticated machine learning frameworks holds promise for developing robust, individualised predictive models that not only identify at‐risk soldiers but also inform the design of personalised preventive strategies.

### Interdisciplinary integration and scalable intervention programmes

Addressing the complex mental health challenges posed by UAV‐related PTSD necessitates an interdisciplinary approach synthesising insights from neurobiology, epigenetics and clinical psychology to develop innovative, mechanism‐based interventions. Recent research highlights the importance of targeting specific molecular pathways and neural circuits implicated in PTSD pathophysiology, such as those modulated by dysregulated microRNAs affecting BDNF and inflammatory signalling pathways.^67^ Translating these neurobiological findings into clinical practice requires collaboration among psychiatrists, neurologists, pain specialists and military leadership to establish comprehensive management frameworks that encompass the biological, psychological and social dimensions of care.

Given the potentially large scale of affected UAV operator populations—as evidenced by studies in conflict zones such as Ukraine—there is an urgent need to develop scalable and accessible intervention models. Technology‐enabled remote therapies, including virtual reality‐based exposure treatments and telepsychology platforms, offer promising avenues for delivering evidence‐based care to dispersed military personnel.^68^ Additionally, stepped‐care models that allocate resources efficiently based on symptom severity and risk profiles can optimise treatment delivery while maintaining quality. Peer support programmes, leveraging military culture camaraderie, can further augment formal interventions by providing social connectedness and reducing stigma.^69^

Establishing multidisciplinary teams that integrate expertise from psychiatry, neurology, pain medicine, ethics and military command structures is critical to implementing comprehensive care models. Such teams can ensure interventions are ethically sound, culturally sensitive and operationally feasible within military contexts. Furthermore, ongoing evaluation and adaptation of intervention programmes based on emerging scientific evidence and service member feedback will enhance their effectiveness and sustainability. Future research should prioritise the ‘other side’ of drone warfare—systematically studying the psychological consequences for ground forces, civilians and entire communities exposed to drone operations, including phenomena such as persistent helplessness, collective trauma and the ‘fear of the sky’ (lithophobia).

## CONCLUSION

The integration of UAVs into modern warfare has undeniably transformed combat operations, yet it has concurrently introduced unprecedented psychological challenges for military personnel. From an expert perspective, the emergence of UAV‐related PTSD represents a complex and multifaceted phenomenon demanding nuanced understanding and balanced approaches to research and clinical management. This review has synthesised current knowledge across neurobiological, epigenetic, clinical and therapeutic domains, highlighting the intricate interplay between the unique operational environment of UAVs and the mental health outcomes of their operators and targets.

One of the most striking insights is the identification of distinct neurobiological and epigenetic signatures associated with UAV‐related PTSD. Unlike traditional combat‐related PTSD, which often stems from direct physical threat and sensory immersion, UAV operators experience psychological trauma characterised by paradoxical detachment from the battlefield coupled with intense moral and cognitive burdens. Neuroimaging studies reveal prefrontal cortex function alterations, implicating deficits in executive control and emotional regulation. Concurrently, inflammatory pathways appear activated, and specific DNA methylation patterns have been observed, suggesting that UAV‐related PTSD is not only a psychological condition but also a disorder with tangible molecular and structural brain correlates.

However, the clinical presentation of UAV‐related PTSD is further complicated by frequent comorbidities such as chronic pain, cognitive impairments, dissociative symptoms and substance use disorders. These overlapping conditions challenge traditional diagnostic criteria and therapeutic protocols, which were primarily designed for conventional combat trauma. Moreover, the phenomenon of digital moral trauma—stemming from the ethical dilemmas and perceived responsibility inherent in remote warfare, amplified by technological mediation—adds a layer of psychological complexity that standard PTSD treatments may inadequately address. The low engagement rates in conventional therapies among UAV operators, often due to intentional avoidance and stigma, highlight the urgent need for adaptive interventions that are sensitive to the unique experiential and moral context of this population.

Balancing these diverse research perspectives requires a multidisciplinary approach respecting the biological underpinnings of UAV‐related PTSD while acknowledging its psychosocial and ethical dimensions. Future research must prioritise developing multimodal risk assessment models that integrate neuroimaging, epigenetic markers, psychological profiling and operational data to identify individuals at heightened risk prior to symptom onset. Such predictive tools would enable early, targeted interventions and potentially mitigate progression to chronic PTSD. Additionally, intervention strategies should be scalable and incorporate neuroscientific insights, such as neurofeedback, anti‐inflammatory treatments and epigenetic modulators, alongside psychotherapeutic modalities tailored to address moral trauma and avoidance behaviours.

Furthermore, establishing comprehensive, interdisciplinary management frameworks is essential. These frameworks should facilitate collaboration among neuroscientists, clinicians, ethicists, military leadership and mental health specialists to design and implement prevention and treatment programmes that are both evidence‐based and contextually relevant. Training programmes that enhance resilience and ethical decision‐making, combined with accessible mental health services that reduce stigma and encourage engagement, will be critical components.

In conclusion, the advent of UAV technology in warfare has precipitated a paradigm shift in the nature of combat‐related psychological trauma. UAV‐related PTSD represents a complex clinical entity characterised by unique neurobiological alterations, epigenetic modifications and challenging comorbidities, further complicated by moral trauma and barriers to treatment engagement. Addressing these challenges requires a balanced, expert‐driven synthesis of diverse research findings and clinical insights. By advancing precision risk assessment, developing integrative and adaptive interventions and fostering interdisciplinary collaboration, military and medical communities can better safeguard the mental health and operational readiness of UAV personnel. This holistic approach not only promises improved outcomes for affected individuals but also contributes to a broader understanding of trauma in the evolving landscape of modern warfare.

## AUTHOR CONTRIBUTIONS

Conceptualization: Dawei Zhang and Chenglin Sang. Data analysis: Yu Xue, Yongliang Zhang. Bioinformatics validation: Yongxian Zhang. Manuscript drafting: Yu Xue, Yufang Sun and Dawei Zhang. Manuscript revision and supervision: Dawei Zhang and Yongxian Zhang.

## FUNDING

The authors have nothing to report.

## CONFLICT OF INTEREST STATEMENT

The authors declare no conflicts of interest.

## ETHICS STATEMENT

Not applicable.

## Data Availability

The authors have nothing to report.
